# Damned if you do, damned if you don't: contextual decision-making around SOGI data collection in the Deep South

**DOI:** 10.3389/fpubh.2026.1800338

**Published:** 2026-05-29

**Authors:** Sarah MacCarthy, Peter Nguyen, Mallie Froehlich, Jane Carol Murphy, Gabe H. Miller, Nicholas Carlisle

**Affiliations:** 1 The University of Alabama, Birmingham, AL, United States; 2 RAND Corporation, Santa Monica, CA, United States; 3 Tulane University, New Orleans, LA, United States

**Keywords:** Deep South, gender identity, harm reduction, minority stress, sexual orientation, SOGI data, structural determinants

## Abstract

Sexual orientation and gender identity (SOGI) data collection is often framed as a cornerstone of diversity, equity, inclusion, and accessibility efforts. Yet decisions about whether, how, and when to collect SOGI data are inherently contextual and increasingly precarious. Drawing on prior empirical synthesis and lived experience navigating the termination of federally funded sexual and gender minority (SGM)-focused grants in the United States (U.S.), this Perspective argues that SOGI data systems can function as structural determinants of visibility, safety, and wellbeing for SGM populations. We synthesize what the evidence suggested prior to recent policy shifts and examine how successive grant terminations–beginning with a U.S. National Science Foundation-funded project and later extending to National Institutes of Health training mechanisms supporting doctoral students–signal a broader erosion of institutional protections. Importantly, we argue that heightened risk does not necessitate data abandonment; rather, it asks us to understand how constraint reshapes our obligation. We propose a harm-reduction framework that emphasizes proportionality, methodological creativity, and adaptive safeguards, enabling equity-oriented evidence generation while minimizing harm under restrictive U.S. geographic, institutional, and community conditions.

## Introduction

This Perspective did not originate as a theoretical exercise. It emerged from the collapse of multiple federally funded projects examining sexual and gender minority (SGM) workforce diversity and health equity. In 2023, a U.S. National Science Foundation (NSF)-funded project focused on SGM representation in science, technology, engineering and mathematics (STEM) was terminated after being deemed too controversial. The termination occurred mid-project, leaving staff positions unfunded and placing them in immediate jeopardy. At the time, this was experienced as an isolated incident rather than part of a broader pattern.

In hindsight, it was an early warning. In 2025, this pattern intensified nationally across the U.S. Within a short period, nearly an entire portfolio of our SGM-focused

grants was terminated, including training mechanisms that supported pre-doctoral fellows. The consequences were not abstract. Fellows faced the loss of stipends, tuition coverage, and health insurance. Staff faced sudden unemployment. Community partners faced the withdrawal of promised support. Considerable effort shifted from research and practice to crisis management: securing bridge funding, fundraising to maintain salaries and benefits, renegotiating institutional commitments, and shielding trainees from the downstream effects of political decisions over which they had no control.

These actions were not specific to the U.S. but were instead part of a broader global movement undermining evidence-based medicine and clinical guidelines. Across Europe, a growing wave of similar actions emerged: in 2020, Finland introduced guidelines restricting initiation of hormone therapy to narrowly defined cases of severe dysphoria (1); in 2022, Sweden limited puberty blockers and hormone therapy to “exceptional cases” (2); and that same year, France issued guidance urging heightened medical caution while still permitting treatment with parental consent (3). In 2024, the United Kingdom National Health Service restricted access to puberty blockers to research settings only (4). Other countries across the globe have also followed suit. For example, in Brazil, the national regulatory body for physicians issued a resolution significantly limiting the use of puberty blockers–one that ultimately was suspended several months later by a federal court (5). While these developments reflect the broader global context, the analysis that follows is grounded in our experiences within the U.S., and specifically in the Deep South, where shifts in funding, policy, and institutional practice have shaped how we collect and use SOGI data.

In our experience, despite these disruptions, research and practice continued. Staff were retained, fellows were supported, and partnerships were sustained through a patchwork of institutional resources, philanthropy, and community investment. Within the U.S. context, these experiences fundamentally reshaped our understanding of risk, responsibility, and the obligations associated with collecting and stewarding SOGI data. They provide the context from which we revisit earlier empirical work on SOGI data collection in our workplaces.

## What the evidence told us then: the pearls

Our scoping review, initially commissioned by NSF in 2022 and later updated in 2024, revealed a striking absence of empirical literature on workplace SOGI data collection. The U.S. federal government was the only large employer systematically collecting SOGI data, enabling limited estimates suggesting substantial underrepresentation of SGM individuals in STEM (6). Beyond these few studies, the literature focused largely on disclosure dynamics and workplace climate rather than formal measurement.

Across studies, disclosure was frequently associated with improved collegial relationships, increased access to mentorship, and greater comfort at work (7–10). Concealment, by contrast, was linked to anxiety, burnout, and diminished mental health—patterns consistent with minority stress theory (7, 10, 11). These benefits were not universal, however. Some studies found no association between disclosure and job satisfaction, while others observed higher turnover among SGM employees even in ostensibly inclusive environments (12, 13). Gender minority employees consistently faced heightened risks related to disclosure, reflecting differential exposure to stigma, surveillance, and institutional vulnerability (8, 9, 14). Collectively, the evidence suggested that disclosure–and by extension SOGI data collection–was most likely to be beneficial when embedded within protective policies, inclusive cultures, and enforceable safeguards (15–22). Even at the time, these conditions were unevenly distributed, particularly in of conservative regions (23–25). These findings, however, were not published following the termination of our NSF contract.

## What the evidence means now: the perils

Subsequent political and policy developments fundamentally altered the landscape in which SOGI data collection occurs. Beginning in 2024, a wave of state actions targeted our SGM-related research, diversity initiatives, and related programs. The termination of training grants in 2025, including mechanisms supporting fellows and early-career investigators, marked a shift from localized, state-level controversy to systemic change at the federal level.

In this environment, SOGI data collection–once promoted as a best practice–became a potential site of risk. Questions emerged regarding subpoena exposure, compelled disclosure, data misuse, as well as professional repercussions for investigators and our community members. The ethics of measurement could no longer be disentangled from the political context in which data are generated and stored.

## A harm-reduction framework for contextual decision-making

In response, we advance a harm-reduction framework for SOGI data decision-making. Harm reduction, widely applied in public health, prioritizes minimizing potential harms while recognizing that ideal conditions are not always attainable. Importantly, harm reduction does not imply inaction. Rather, it reframes SOGI data collection as an adaptive process that evolves in response to changing risk environments rather than defaulting to either universal adoption or abandonment.

Within this framework, affirmative responses to contextual risk indicators–such as operating in a punitive environment, working within a state institution with policies restricting diversity, equity, inclusion, and accessibility (DEIA) efforts, or serving communities experiencing heightened fear–do not constitute a rationale to cease data collection altogether. Instead, they signal the need for greater methodological creativity, deeper integration across approaches, and more intentional safeguards.

First, geographic context must be assessed. Legal protections, public records laws, and enforcement practices vary widely. In regions such as the Deep South, where protections may be limited or actively eroding, collecting identifiable SOGI data may increase risk. Harm reduction in these settings emphasizes *how* data are collected rather than *whether* they are collected–prioritizing approaches that reduce identifiability, distribute risk, and limit downstream exposure. For example, in higher-risk settings, standard practices such as aggregation and de-identification become essential safeguards given the potential for data to be subpoenaed (26). These conditions also challenge the default pursuit of “ideal” longitudinal data, as repeated follow-up often requires maintaining identifiable information. In turn, researchers must more deliberately weigh the benefits of richer data against the risks imposed on participants, revisiting the principle of beneficence not as a theoretical norm but as a practical constraint guiding what data should be collected at all.

Second, institutional context shapes both constraint and possibility. Public universities and agencies may be bound by restrictive state laws and funding dependencies, while private or federal institutions may retain greater autonomy–though in practice, they frequently remain more accountable to state governments than is widely assumed (27). Yet constraint can also prompt innovation. In our own experience, the closure of traditional pathways for data collection forced a more systematic integration of previously siloed approaches–embedding SOGI-related inquiry across programs, methods, and partnerships rather than concentrating it in any single, easily available mechanism. In parallel, these limitations invite more creative engagement with retrospective data–which can be useful, even if incomplete. While such data cannot fully capture the effects of the current policy environment, partnering with community organizations and clinical systems to examine existing electronic health records that include SOGI measures offers a way to assess, in near real time, how broader structural shifts are shaping health outcomes.

Third, community context must be centered. SGM populations do not experience risk uniformly, and voluntariness is often shaped by power asymmetries. Meaningful harm reduction requires engaging communities not only as data sources, but as partners in determining acceptable levels of risk and appropriate modes of inquiry. In practice, this means embedding community perspectives across all stages of research–from study design and question formulation to implementation, interpretation, and dissemination–rather than confining input to advisory roles (28). It also requires expanding how institutions value and resource this expertise, including creating paid, decision-making roles for community members within formal governance structures such as Institutional Review Boards, rather than relying solely on community advisory boards. These shifts move engagement from symbolic inclusion to shared authority, strengthening both the ethical integrity and real-world relevance of research conducted in constrained environments.

Operationally, harm-reduction strategies may include anonymized or aggregate data collection; opt-in rather than mandatory disclosure; separation of identity data from personnel, training, or evaluation records; restricted access to trained personnel; enhanced encryption; or the strategic redistribution of data collection across time, settings, or methods. In some contexts, qualitative, community-engaged, or mixed-method approaches may provide safer and more resilient pathways for documenting inequity.

While trainees and fellows may face elevated vulnerability due to reliance on institutional support for income, health insurance, and professional advancement, they often have the greatest insights regarding a path forward. One student described this adaptive process as akin to gardening: when the visible growth is repeatedly cut back, the roots are forced to grow deeper. Under conditions of constraint, harm reduction does not eliminate growth; it changes where and how that growth occurs. In this sense, contextual decision-making is not about retreat, but about cultivating durability in the face of pressure.

## Conclusion

In the U.S., the absence of SOGI data continues to constrain efforts to document inequities and design responsive interventions. At the same time, the cascading termination of SGM-focused research and training grants underscores the fragility of the infrastructures that once made such data collection feasible. These dynamics are not abstract to us–they are grounded in our experiences working in the U.S. Deep South, where shifts in priorities have had immediate and material consequences for our researchers, trainees, and community partners. While this Perspective is grounded in this context, we invite researchers and practitioners across other counties and countries to consider whether and how this framework resonates within their own environments–and where it may require adaptation given differing landscapes. The task before public health, here and likely elsewhere, is not to declare SOGI data collection universally good or bad, but to recognize it as a situated decision shaped by place, power, and risk.

By adopting a harm-reduction approach grounded in context, institutions can better navigate the tension between visibility and safety, thereby protecting individuals, sustaining trainees and fellows, and preserving the capacity to produce equity-oriented evidence even under conditions of constraint.

When funding infrastructures collapse, so too do the safeguards that make equity-focused data collection possible, making adaptive, context-responsive harm-reduction strategies not optional, but essential.

## Data Availability

The raw data supporting the conclusions of this article will be made available by the authors, without undue reservation.
